# Dermatology-Related Quality of Life Measured with the Dermatology Life Quality Index in Basal Cell Carcinoma and Other Non-Melanoma Skin Cancers: A Systematic Review and Exploratory Meta-Analysis

**DOI:** 10.3390/jcm15166351

**Published:** 2026-08-17

**Authors:** Jakub Nicer, Justyna Tomaszewska, Dariusz Jurkiewicz, Piotr Rot, Maria Sobol

**Affiliations:** 1 Department of Otolaryngology and Oncological Otolaryngology with Clinical Department of Cranio-Maxillofacial Surgery, Military Institute of Medicine—National Research Institute, 04-141 Warsaw, Poland; nicer35@gmail.com (J.N.); djurkiewicz@wim.mil.pl (D.J.); rot@autograf.pl (P.R.); 2 Institute of Mathematics and Cryptology, Military University of Technology, 2 Kaliskiego Str., 00-908 Warsaw, Poland; justyna.tomaszewska@wat.edu.pl; 3 Department of Biophysics, Physiology and Pathophysiology, Medical University of Warsaw, 5 Chałubińskiego Str., 02-004 Warsaw, Poland

**Keywords:** basal cell carcinoma (BCC), dermatology life quality index (DLQI), meta-analysis, non-melanoma skin cancer (NMSC)

## Abstract

**Background/Objectives:** Basal cell carcinoma (BCC) is the most common skin cancer and may negatively influence a patient’s well-being despite low mortality. This systematic review and exploratory meta-analysis evaluated dermatology-related quality of life, as measured exclusively by the Dermatology Life Quality Index (DLQI), in patients with BCC and other non-melanoma skin cancers (NMSC). Instruments other than the DLQI, including disease-specific measures of appearance, scarring, fear of recurrence and treatment satisfaction, were outside the scope of the quantitative synthesis. **Methods:** A systematic search of the PubMed, Scopus, and the Web of Science was conducted until 5 April 2026 following the PRISMA guidelines. Studies reporting DLQI outcomes in adult patients with BCC, squamous cell carcinoma (SCC), or NMSC were included. Pooled baseline DLQI scores and pre- to post-treatment changes were calculated using a random effects model. **Results:** Six studies were included. The pooled baseline DLQI score was 3.76 (95% CI: 2.18–5.33) in the BCC-only analysis and 4.07 (95% CI: 2.99–5.15) in an exploratory expanded analysis including SCC and broader NMSC populations; this estimate should be interpreted as a summary of heterogeneous keratinocyte cancer populations rather than as representative of BCC alone. None of the included studies had an untreated comparator group, so the pre–post results describe the change in DLQI score following treatment rather than a treatment effect. The pooled reduction was 2.38 points (95% CI: 0.56–4.19) in the BCC-only and 1.90 points (95% CI: 0.84–2.96) in the expanded pre–post analysis. Both fall below the minimal clinically important difference for the DLQI of approximately four points. Heterogeneity was very high throughout (I^2^ > 95% for baseline analyses), so the pooled means summarize highly dispersed evidence and cannot be read as representative average values. Surgical treatment appeared to show a greater improvement in DLQI scores compared with the single available radiotherapy cohort. This comparison should be interpreted descriptively due to the inclusion of only one radiotherapy study. **Conclusions:** Patients with BCC and other NMSCs experience measurable impairment in dermatology-related quality of life before treatment, although the overall burden is generally mild. Treatment is associated with improvement in DLQI scores; however, the magnitude of change is modest and may not consistently exceed thresholds considered clinically meaningful. Evidence regarding differences between treatment modalities remains limited, particularly for radiotherapy, and further prospective studies using disease-specific quality-of-life instruments are needed.

## 1. Introduction

Basal cell carcinoma (BCC) is the most common type of skin cancer, particularly in white-skinned populations in Europe, North America and Australia [1,2]. BCC accounts for approximately 70–90% of all skin cancers [3,4]. Due to aging populations and more optimal diagnosis, incidence rates of BCC and other ultraviolet-related skin cancers are expected to increase [3]. In Europe, BCC incidence rates have risen by approximately 5.5% over the last decade [5]. In the USA, the increase in incidence rates varies between 2 and 8% [1].

BCC is a slow-growing malignant tumor that is most often treated by surgical excision, although topical therapies, radiotherapy, and other nonsurgical approaches may be appropriate in selected cases [3,5]. Mohs micrographic surgery (MMS) is commonly used for high-risk tumors because it enables complete microscopic assessment of surgical margins while preserving as much healthy tissue as possible [6,7].

Mohs micrographic surgery is generally recommended for high-risk basal cell carcinomas and tumors located in cosmetically and functionally sensitive areas because it allows complete margin assessment while preserving healthy tissue [2,8,9]. Standard surgical excision remains the preferred treatment for most low-risk lesions [10]. Both approaches provide excellent oncologic outcomes, although MMS has been associated with lower recurrence rates in selected high-risk patients [3,10,11,12,13]. The choice between the two approaches is guided by tumor and patient characteristics, as well as by the availability of appropriately trained personnel and the greater time and resource requirements of MMS [14,15].

Basal cell carcinoma, although rarely life-threatening, may substantially impair patients’ quality of life (QoL) due to its frequent involvement of highly visible body areas, particularly the skin of the face and head. As the skin represents both the largest organ and a central component of personal and social identity, dermatologic malignancies may affect self-perception, body image, and psychosocial well-being. Cancer diagnoses have been associated with a range of adverse psychological outcomes, including anxiety, depression, emotional vulnerability, and social distress [16]. In addition, BCC and its treatment may result in cosmetic disfigurement, financial burden, and limitations in activities of daily living, all of which may contribute to the reduced QoL [17]. The degree of impairment is, however, not uniform and depends strongly on tumor location. Lesions of the central face—including the nose, eyelids, lips and ears, which correspond to the high-risk “H zone”—are among the most clinically consequential because these areas are constantly visible, cannot be concealed by clothing, and contain structures where even small tissue defects may affect function and facial symmetry. Reconstruction in these areas more often requires flaps or grafts rather than primary closure, and the resulting scars, contour changes and occasional functional sequelae such as epiphora or nasal valve narrowing may contribute substantially to patient distress. Truncal and limb lesions, by contrast, are typically excised with simple closure and are concealed in everyday life, and are correspondingly associated with lower reported impairment. The burden is also cumulative rather than tied to a single lesion. This reflects the underlying process of chronic ultraviolet-induced field cancerization: the same photodamaged skin that gives rise to one keratinocyte cancer predisposes to subsequent lesions. Consequently, many patients require lifelong surveillance, repeated treatments and continuous photoprotection, meaning that the impact of keratinocyte cancer extends beyond the individual tumor and contributes to long-term impairment in quality of life [8,18]. This cumulative burden is particularly evident in patients who develop multiple keratinocyte cancers, including, in rare cases, those with syndromic conditions such as basal cell naevus (Gorlin–Goltz) syndrome, in whom repeated treatments, progressive scarring and lifelong surveillance may substantially affect quality of life. Consequently, QoL has emerged as an important patient-reported outcome in oncologic and dermatologic research.

Several questionnaires are available to assess quality of life in dermatology, with the Dermatology Life Quality Index (DLQI) being one of the most widely used. The DLQI has been validated in many inflammatory and neoplastic skin diseases and is commonly used in both clinical practice and research, allowing results to be compared across different patient populations. It includes 10 questions covering symptoms, daily activities, leisure, work or school, personal relationships, and treatment during the previous week. Each question is scored from 0 (“not at all”) to 3 (“very much”), giving a total score from 0 to 30, where higher scores indicate greater impairment in quality of life. The quality-of-life burden of keratinocyte cancer must also be understood against the background of its principal cause. Cumulative ultraviolet radiation drives skin carcinogenesis through direct DNA photoproduct formation, oxidative stress and local immunosuppression, and the same chronically photodamaged field that gives rise to one tumor predisposes the patient to further lesions, so that patients are typically managed for a field of disease rather than for an isolated tumor. This has direct implications for quality of life: it accounts for the visible actinic changes and dyspigmentation that persist irrespective of treatment success, for the need for indefinite surveillance and photoprotection, and for the anxiety associated with the recognition that further lesions are likely. Non-melanoma skin cancer has accordingly been described as imposing a burden that extends beyond the individual tumor [18].

Although the DLQI is widely used in dermatology, relatively few studies have focused on patients with BCC and other non-melanoma skin cancers. Existing studies suggest that these tumors can impair quality of life, particularly when they affect visible areas of the face or require more extensive treatment. Most have also reported improvements in DLQI scores after treatment, but the findings have varied because of differences in study design, patient characteristics, treatment modalities, and follow-up. As a result, the overall impact of NMSC on quality of life and the extent of improvement after treatment remain uncertain, providing the rationale for the present systematic review and exploratory meta-analysis [19,20,21,22,23,24].

The aim of this systematic review and exploratory meta-analysis was to summarize available evidence on DLQI outcomes in patients with BCC, SCC, and mixed NMSC populations. Specifically, we aimed to estimate the baseline DLQI impairment, assess changes in DLQI scores following treatment, and identify limitations in the current research evidence relevant to future patient-reported outcome research in keratinocyte cancers.

## 2. Materials and Methods

Studies included in this research were identified through a systematic literature search of the PubMed, Scopus, and Web of Science databases. All stages of study selection, data extraction, and data verification were conducted independently by two human reviewers (M.S. and J.T.). Records retrieved from the three databases were exported to a reference manager, in which duplicates were removed automatically by matching digital object identifiers (DOIs) and titles and subsequently verified manually; 283 duplicate records were excluded before screening. The remaining records were exported to Microsoft Excel (Microsoft 365 (Microsoft Corporation, Redmond, WA, USA)), where titles and abstracts were screened independently using a standardized screening spreadsheet. Potentially eligible studies subsequently underwent independent full-text assessment by the same reviewers. Data were extracted independently by M.S. and J.T. using a piloted standardized Microsoft Excel data extraction form. The extracted data were cross-checked against the original publications to verify their accuracy and completeness. Any discrepancies identified during study selection or data extraction were resolved through discussion and critical re-evaluation until consensus was reached; therefore, no third reviewer was required. No automation tools or artificial intelligence-assisted software were used during the screening or data extraction process, and the authors of the included studies were not contacted to obtain additional data. No restrictions regarding the date of publication were applied during the search process. Consequently, all records available in the databases from their inception until the final search date were considered. Articles published until and including 5 April 2026 were eligible for inclusion. The inclusion criteria were as follows: original research articles, prospective studies, randomized or cross-sectional studies, human studies, and publications in the English language. Restricting eligibility to English-language reports may have introduced language bias, and relevant studies published in other languages may have been missed; this is acknowledged as a limitation. The following strategy was used for PubMed and adapted to the syntax of Scopus and the Web of Science: (“basal cell carcinoma”[MeSH Terms] OR “basal cell carcinoma”[tiab] OR BCC[tiab] OR “non-melanoma skin cancer”[tiab] OR NMSC[tiab] OR “carcinoma, squamous cell”[MeSH Terms] OR “squamous cell carcinoma”[tiab] OR SCC[tiab]) AND (“quality of life”[MeSH Terms] OR “quality of life”[tiab] OR QoL[tiab] OR “Dermatology Life Quality Index”[tiab] OR DLQI[tiab]). Each database was searched on 5 April 2026. In addition, the reference lists of all included studies and of relevant reviews were screened by hand. The complete search strings for all three databases are provided in the Supplementary Materials (Table S1).

**Figure 1:**
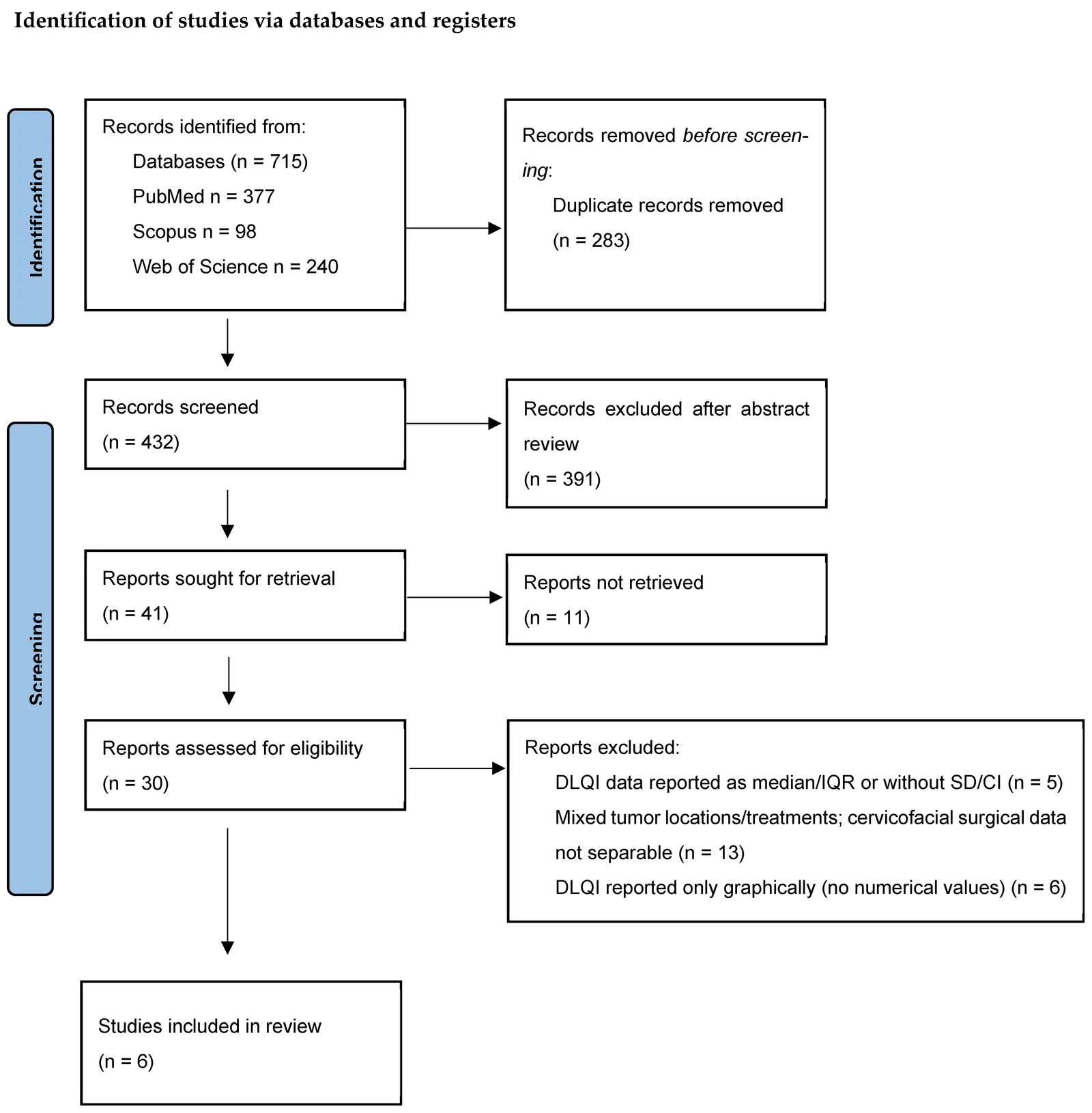
Flow chart for the inclusion of studies.

For the analysis, studies were excluded if they (1) reported DLQI data only as the median and the range or the median and the interquartile range (IQR) or they reported the DLQI without the standard deviation (SD) or confidence interval (CI), (2) usedvarious location and treatment methods when the cervicofacial location and surgical treatment could not be distinguished, or (3) expressed the DLQI as graphs, without values (Figure 1). Case reports, unpublished studies, and conference abstracts were not considered, and the authors of the included studies were not contacted for additional data.

Two reviewers (MS and JT) independently screened the titles, abstracts, and full-text articles for eligibility based on predefined inclusion and exclusion criteria. Any discrepancies were resolved through discussion and critical reevaluation of the studies until full consensus was achieved. No involvement of a third reviewer was necessary. Only studies for which agreement was reached were included in the final review.

The following data were extracted from each study: the authors, year of publication, number of participants, sex of participants, entry age, Dermatology Life Quality Index scores before and after treatment, the duration of the follow-up, technique used, and the study site.

The systematic review was conducted following the PRISMA guidelines [25] but was not prospectively registered in PROSPERO or any comparable registry, and no pre-registered protocol was published; this is acknowledged as a limitation. Specific requirements are listed below.

### 2.1. Study Group

Inclusion criteria: The inclusion criteria comprised adult male and female patients, aged ≥16 years, diagnosed clinically and/or histopathologically with basal cell carcinoma (BCC), squamous cell carcinoma (SCC), or non-melanoma skin cancer (NMSC). Eligible participants were required to provide informed consent and possess adequate physical, cognitive, and linguistic abilities to complete validated quality-of-life questionnaires.

Limits used: conducted in human participants and involving patient-reported quality-of-life, studies published in English.

Timing: No restrictions were applied to the start date of the search. All records available from database inception to 5 April 2026 were eligible for inclusion.

### 2.2. Statistical Analysis

Statistical analyses were performed using TIBCO Statistica version 13 (TIBCO Software Inc., Palo Alto, CA, USA). Pooled estimates were calculated using an inverse-variance weighted random-effects model, with the between-study variance (τ^2^) estimated by the DerSimonian–Laird method. Each cohort was weighted by the inverse of its total (within-plus between-study) variance, the within-study variance being SD^2^/*n*. Between-study heterogeneity was assessed using Cochran’s Q test and quantified using the I^2^ statistic, with values of 0–25%, 25–50%, 50–75%, and >75% indicating negligible, low, moderate, and high heterogeneity, respectively. The between-study variance (τ^2^) was estimated separately for each subgroup in subgroup analyses. Baseline DLQI scores were pooled using study-level mean values and corresponding standard deviations, and pooled estimates with 95% confidence intervals (CIs) were calculated.

For the pre–post treatment analysis, mean DLQI scores before and after treatment were extracted from each study. Because the correlation between pre- and post-treatment measurements was not reported in the included studies, the standard deviation of the change score was estimated using the following formula:

$$SD=\sqrt{{SD}_{pre}^{2}+{SD}_{pos}^{2}-2r\cdot {SD}_{pre}\cdot {SD}_{post}}$$

where *r* represents the assumed within-patient correlation coefficient. The primary analysis used an assumed correlation of *r* = 0.50. Sensitivity analyses were subsequently performed using alternative correlation coefficients (*r* = 0.25 and *r* = 0.75) to examine how far the pooled estimates depend on this assumption (Table 1). It must be emphasized that, because r was assumed rather than observed, the change-score standard deviations and hence all confidence intervals and *p*-values for the pre–post analyses are conditional on this assumption and are not fully empirical quantities; they should be read together with the sensitivity analysis in Table 1. In the primary pre–post analysis, the baseline sample size was used to compute the variance of the change score, because the number of patients completing follow-up was not reported consistently across studies. Because this may underestimate uncertainty where attrition occurred, the analysis was repeated using the actual follow-up sample sizes wherever these were reported (Rhee et al., *n* = 101 of 121; Blackford et al., *n* = 34 of 44).

When multiple clinically distinct cohorts were reported within a single study, they were extracted separately because the patient populations were independent and provided relevant disease-specific information. However, because multiple effect estimates originated from the same publication, some degree of within-study methodological dependence cannot be excluded. This applies in particular to Abedini et al. [22], which contributes three separate cohorts (BCC, SCC and combined BCC/SCC) to the expanded baseline analysis, and to Çetinarslan et al. [20], which contributes both a BCC and an SCC cohort. Because these cohorts share investigators, setting, recruitment period and measurement procedures, treating them as statistically independent understates the true correlation between them and therefore artificially narrows the confidence intervals and overstates the precision of the pooled estimates. Due to the limited number of included studies and the insufficient number of independent effect estimates, multilevel meta-analysis or cluster-robust variance estimation could not be reliably performed. Therefore, these findings should be interpreted as exploratory, and the precision of pooled estimates may be overestimated.

The certainty of the body of evidence for each outcome was assessed using the GRADE approach. Because all included studies were observational, the certainty of the evidence initially started at low and was subsequently evaluated across the five GRADE domains: risk of bias, inconsistency, indirectness, imprecision, and publication bias. Each domain was judged as not serious, serious, or very serious, with the certainty of the evidence downgraded by one level for a serious concern and by two levels for a very serious concern. The GRADE assessment was performed independently by two authors, and any disagreements were resolved through discussion until consensus was reached. The resulting certainty ratings are presented in Table 2. Sensitivity analyses were performed using a leave-one-out approach. All statistical tests were two-sided, and *p* < 0.05 was considered statistically significant.

Publication bias was not formally assessed using funnel plots, Egger’s regression test, Begg’s rank correlation test, or the trim-and-fill method because fewer than ten studies were included in the meta-analysis. Consistent with current methodological recommendations, these methods have limited statistical power and may produce unreliable results when applied to a small number of studies.

The methodological quality and risk of bias of the included studies were assessed independently by two reviewers using the appraisal tool appropriate for each study design. Prospective cohort studies were evaluated using the Joanna Briggs Institute (JBI) Critical Appraisal Checklist for Cohort Studies, whereas cross-sectional studies were assessed using the JBI Critical Appraisal Checklist for Analytical Cross-Sectional Studies. As the study by Finlay et al. [19] was a measurement instrument validation study rather than an observational or interventional study, its methodological quality was assessed using the COSMIN Risk of Bias Checklist, which is specifically designed for assessing the measurement properties of patient-reported outcome measures.

In accordance with JBI guidance, no numerical summary score was calculated. Instead, each checklist item was rated as “Yes,” “No,” “Unclear,” or “Not applicable,” and an overall risk-of-bias judgment was reached by consensus based on a qualitative assessment of the methodological limitations identified within predefined domains considered critical for the present review. These domains included the validity and standardized administration of the DLQI, clarity of the inclusion criteria, completeness of follow-up (where applicable), and appropriateness of the statistical analysis. Studies were classified as having a low risk of bias when the identified methodological limitations were unlikely to substantially influence the validity of the reported findings. A moderate risk of bias classification was assigned when relevant limitations were present but their potential impact on the results was considered limited. Studies were classified as having a high risk of bias when substantial methodological concerns were identified that could have materially affected the reliability or interpretation of the results. Any disagreements between reviewers were resolved through discussion. The results of the risk-of-bias assessment are presented in Table 3 and Table S2.

Because relatively few studies reported DLQI outcomes exclusively in patients with basal cell carcinoma (BCC), the primary analyses were first performed using BCC. To increase the available evidence base, additional analyses subsequently included studies enrolling patients with squamous cell carcinoma (SCC) and mixed non-melanoma skin cancer (NMSC) populations. Separate subgroup analyses were conducted whenever feasible to explore potential disease-specific differences. Accordingly, the pooled estimates should primarily be interpreted as reflecting the overall impact of keratinocyte cancers rather than BCC alone.

## 3. Results

The search strategy identified 377 articles in the PubMed, 98 in Scopus, and 240 in the Web of Science databases. After screening using the phrases “basal cell carcinoma”, “BCC”, “non-melanoma skin cancer”, and “NMSC”, “quality of life”, “QoL”, and “Dermatology Life Quality Index”, “DLQI”, six studies were selected and included [19,20,21,22,23,24]. These studies comprised a total of 548 patients (429 with BCC, 112 with SCC, and 7 with combined or other non-melanoma skin cancers), with individual study sample sizes ranging from 8 to 255 participants. Table 4 summarizes the demographic and study characteristics of the included participants, including age, sex, sample size, and pre- and post-treatment DLQI scores.

### 3.1. Baseline Characteristics

**Figure 2:**
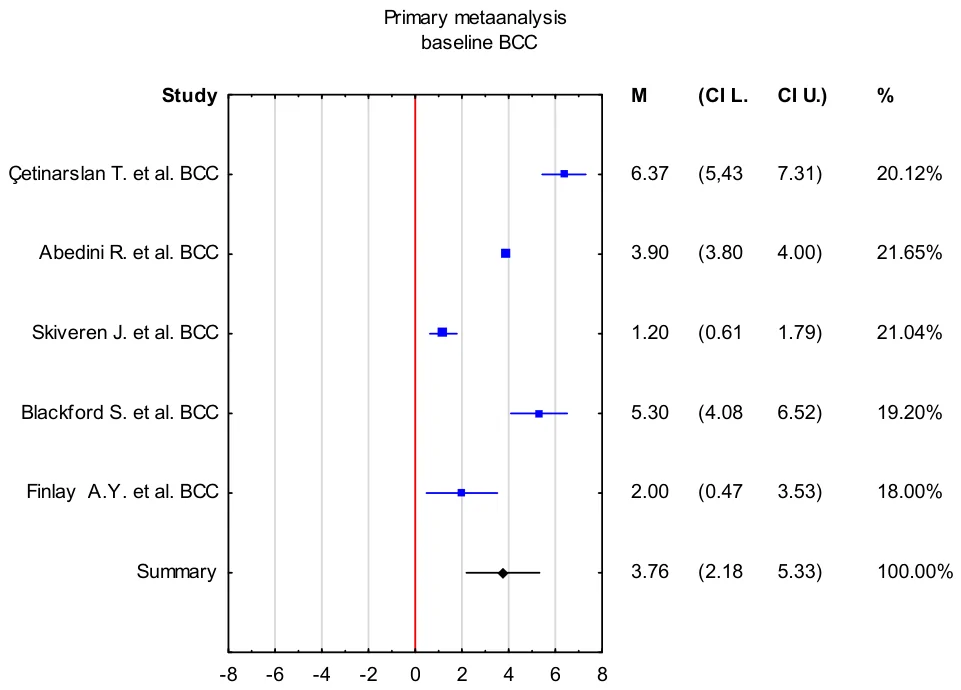
Forest plot of the mean DLQI. Blue squares represent the point estimates for individual studies in the forest plot, with their size proportional to the study’s weight (i.e., its contribution to the summary estimate). Black squares represent the overall summary estimate. M denotes the mean, while CL L. and CL U. denote the lower and upper limits of the confidence interval, respectively. Horizontal lines represent 95% CI [19,20,22,23,24].

For the primary meta-analysis estimating the mean baseline DLQI score in patients with BCC, data from five studies were included: Finlay et al. [19], Çetinarslan et al. [20], Abedini et al. [22], Skiveren et al. [23], and Blackford et al. [24]. The sample sizes of the individual BCC cohorts, one per study, ranged from 8 to 174 patients, involving a total of 326 patients. The overall pooled analysis demonstrated a baseline DLQI score of 3.76 (95% CI: 2.18–5.33). Between-study heterogeneity was very high (I^2^ = 96.6%, τ^2^ = 2.97), supporting the use of a random-effects model (Figure 2).

**Figure 3:**
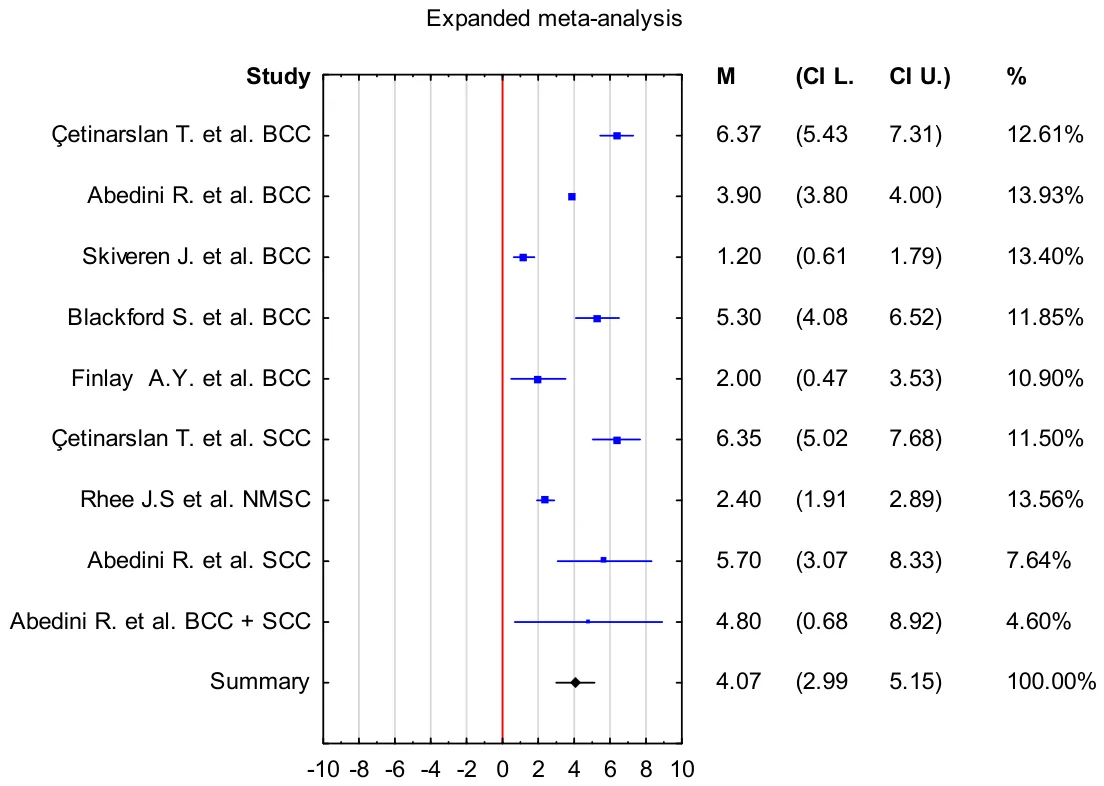
Forest plot of the mean DLQI for expanded meta-analysis. Blue squares represent the point estimates for individual studies in the forest plot, with their size proportional to the study’s weight (i.e., its contribution to the summary estimate). Black squares represent the overall summary estimate. M denotes the mean, while CL L. and CL U. denote the lower and upper limits of the confidence interval, respectively. Horizontal lines represent 95% CI [19,20,21,22,23,24].

To evaluate the influence of broader study populations on the pooled estimate, an expanded meta-analysis was performed by incorporating additional data from studies that also reported DLQI scores for patients with squamous cell carcinoma (SCC), basal cell carcinoma (BCC), and non-melanoma skin cancer (NMSC). This analysis included nine cohorts derived from six studies: five studies contributed a single cohort each, whereas Çetinarslan et al. [20] provided separate BCC and SCC cohorts, and Abedini et al. [22] provided separate BCC, SCC, and combined BCC/SCC cohorts. Overall, the included cohorts comprised 548 participants. Individual cohort sizes ranged from 5 to 174 patients. As some cohorts originated from the same publication (three cohorts from Abedini et al. [22] and two cohorts from Çetinarslan et al. [20]), the nine cohorts were not considered fully independent (Section 2.2). The overall pooled analysis demonstrated a mean baseline DLQI score of 4.07 (95% CI: 2.99–5.15) (Figure 3). High between-study heterogeneity was observed (I^2^ = 95.12%, τ^2^ = 2.18), supporting the use of a random-effects model. The two estimates were numerically close (3.76 for BCC versus 4.07 for the expanded set, a difference of 0.31 DLQI points with widely overlapping confidence intervals), indicating that broadening the population beyond BCC did not materially change the pooled baseline value. It must be stressed, however, that with I^2^ above 95%, almost all of the observed variability arises from differences between studies rather than from sampling error. Under these conditions, a pooled mean does not describe a single underlying value and cannot be read as a representative average DLQI score for these populations; it is reported here only as a summary of a highly dispersed body of evidence, and the range of individual study estimates (1.2 to 6.37) is more informative than the pooled figure itself.

### 3.2. Subgroups Analysis

**Figure 4:**
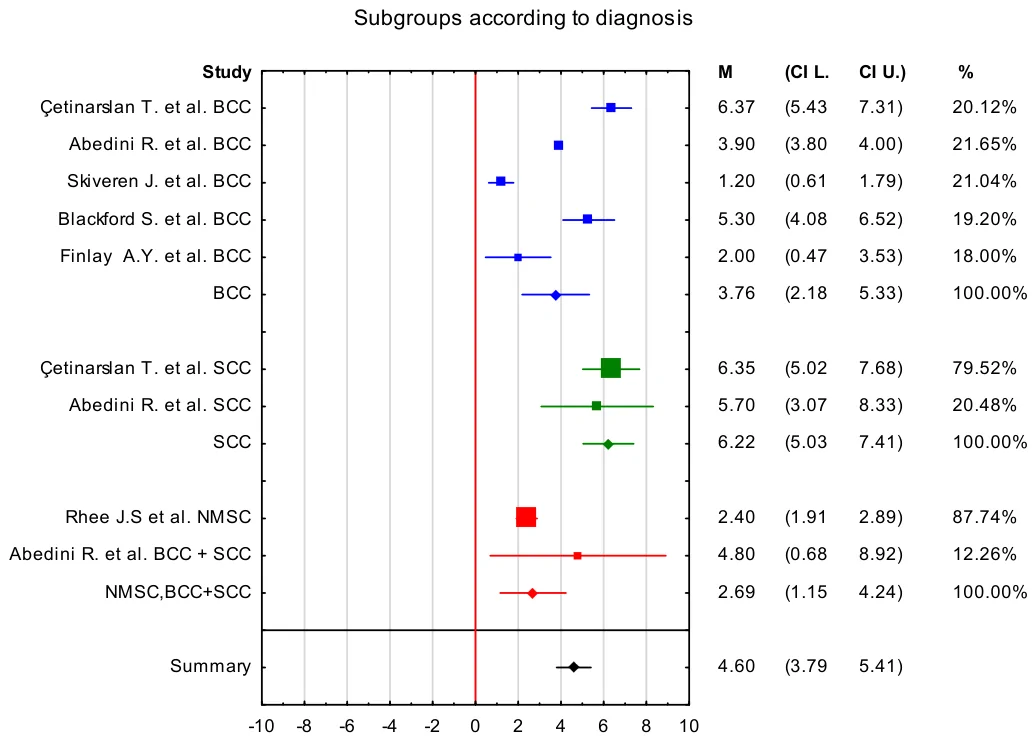
Subgroup analyses stratified by BCC, SCC, mixed BCC/SCC, and NMSC populations. Blue squares represent studies involving patients with BCC, green squares represent studies involving patients with SCC, and red squares represent studies involving patients with combined BCC+SCC or NMSC. The size of each square is proportional to the study’s weight (i.e., its contribution to the summary estimate). Black squares represent the overall summary estimate. M denotes the mean, while CL L. and CL U. denote the lower and upper limits of the confidence interval, respectively. Horizontal lines represent 95% CI [19,20,21,22,23,24].

Since substantial heterogeneity was observed among the included studies, subgroup analyses were performed to explore potential sources of variability in the pooled DLQI estimates. The analyses were stratified according to diagnosis (Figure 4). The pooled mean DLQI score was lowest among patients with NMSC and combined BCC/SCC (2.69, 95% CI: 1.15–4.24), intermediate among patients with BCC (3.76, 95% CI: 2.18–5.33), and highest among patients with SCC (6.22, 95% CI: 5.03–7.41). Although the 95% confidence intervals of the BCC and NMSC/BCC + SCC subgroups partially overlapped, the confidence interval for the SCC subgroup showed little overlap with the other groups, suggesting a greater impairment in quality of life among patients with SCC. The between-group difference was statistically significant (Q_between = 14.074, df = 2, *p* < 0.001), suggesting potential differences in baseline DLQI according to tumor type (Figure 4).

### 3.3. Sensitivity Analysis

**Figure 5:**
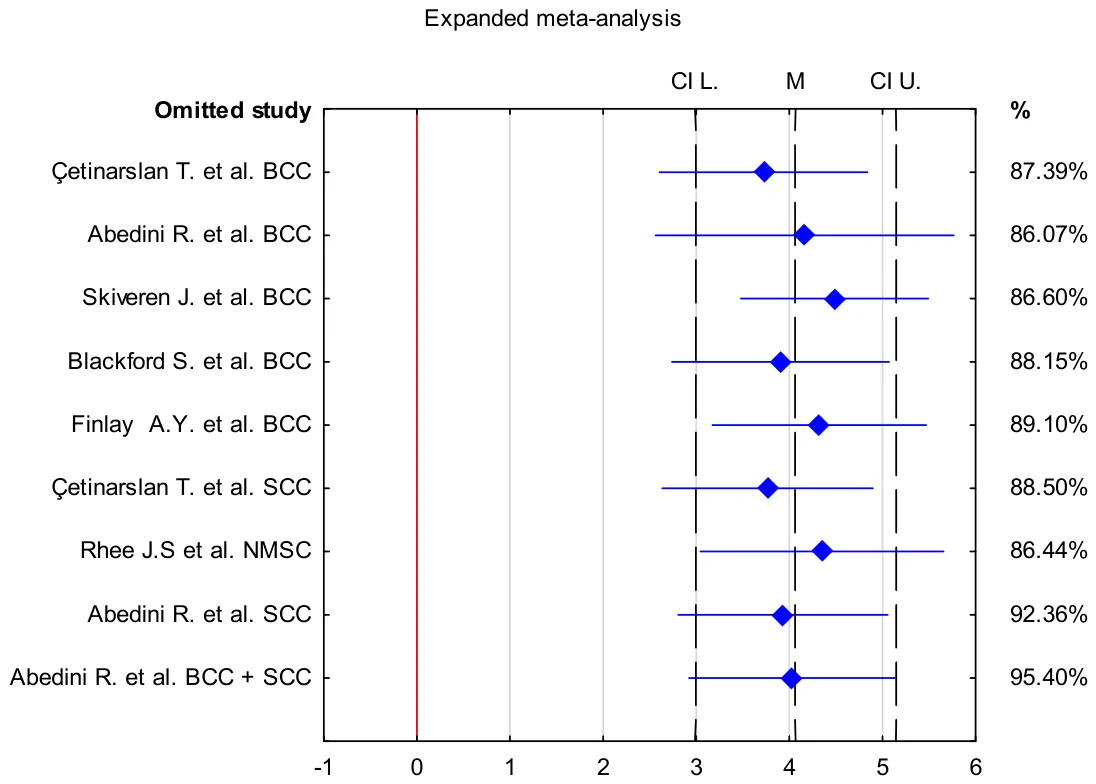
Sensitivity analysis of individual studies on the pooled mean DLQI with 95% confidence intervals. Each study listed on the Y-axis was sequentially omitted to evaluate its impact on the overall pooled estimate. Blue squares represent the point estimate for each study in the forest plot, with their size proportional to the study’s weight (i.e., its contribution to the summary estimate). M denotes the mean, while CL L. and CL U. denote the lower and upper limits of the confidence interval, respectively [19,20,21,22,23,24].

Sensitivity analysis for the baseline model demonstrated minimal variation in pooled estimates after the sequential exclusion of individual studies. Heterogeneity remained consistently high, ranging from 86.07% to 95.40%. The overall pooled baseline estimate remained stable, indicating that no single study excessively influenced the combined effect size (Figure 5).

### 3.4. Pre-and Post-Treatment Effects

The primary meta-analysis included three studies evaluating patients with BCC. The pooled standardized mean change indicated an improvement following treatment, and the sensitivity analyses showed that the direction and approximate magnitude of the change were preserved across the assumed pre–post correlations, although this consistency reflects only insensitivity to that single assumption and does not indicate agreement between studies, which remained poor.

To assess whether the observed treatment effect extended beyond BCC populations, an expanded meta-analysis was performed using the same methodology and including two additional studies involving SCC and NMSC populations. Therefore, the expanded analysis included five studies in total. The pooled effect estimate from the expanded analysis remained consistent with the findings of the BCC-only analysis, indicating improvement after treatment. Sensitivity analyses confirmed that the direction and interpretation of the effect remained stable across the tested correlation assumptions.

Detailed results of the individual studies, pooled effect estimates and sensitivity analyses for both the BCC-only and expanded meta-analyses are presented in Table 2.

**Figure 6:**
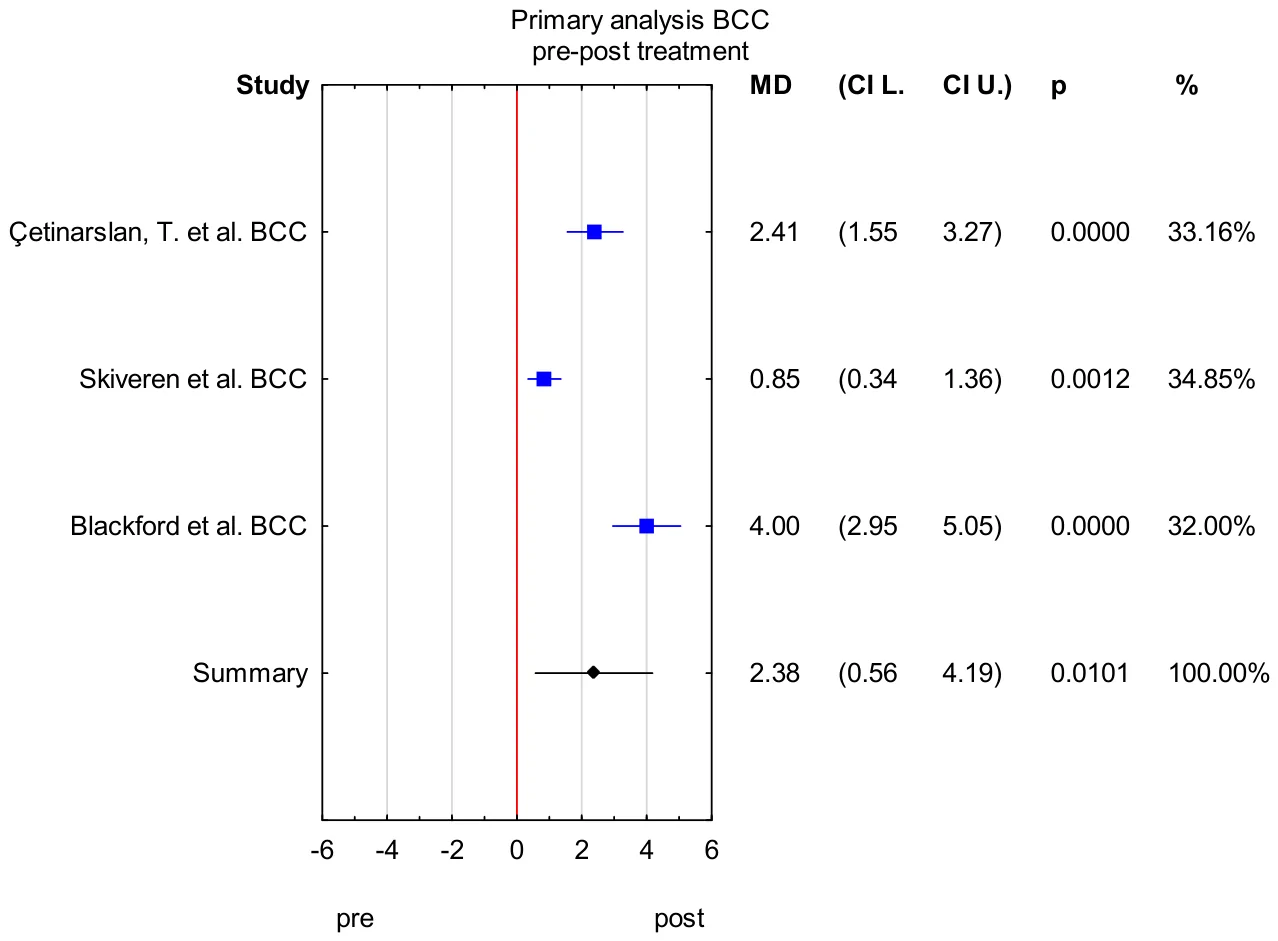
Forest plot of unstratified pre–post treatment change in DLQI scores across all included studies, without adjustment for treatment modality for BCC cohort. The pooled estimate represents the overall mean difference (post-treatment minus baseline), combining surgical and radiotherapeutic cohorts. Blue squares represent the point estimate for each study in the forest plot, with their size proportional to the study’s weight (i.e., its contribution to the summary estimate). Black squares represent the overall summary estimate. MD denotes the mean difference, while CL L. and CL U. denote the lower and upper limits of the confidence interval, respectively, and horizontal lines indicate 95% confidence intervals [20,23,24].

**Figure 7:**
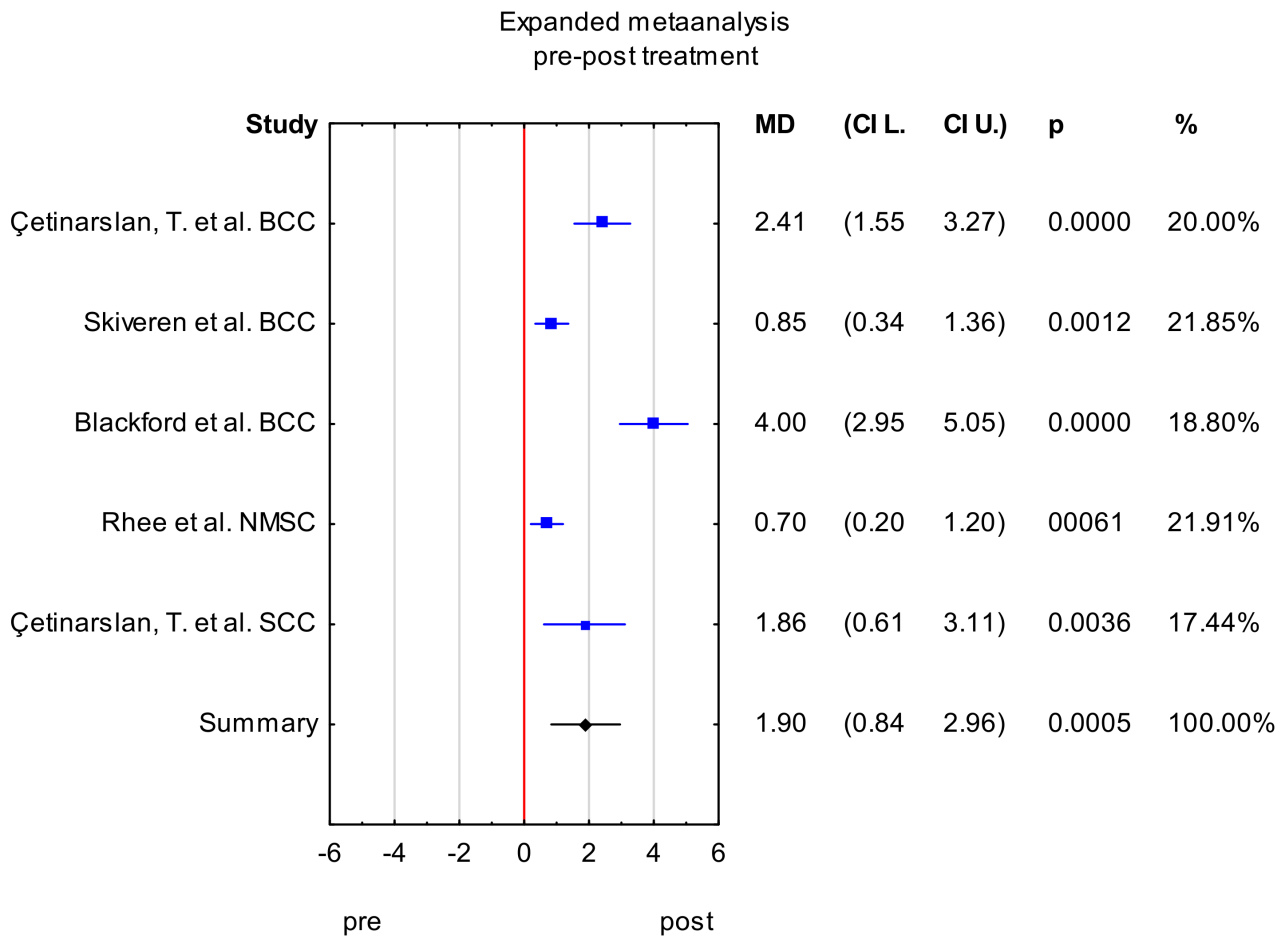
Forest plot of unstratified pre–post treatment change in DLQI scores across all included studies, without adjustment for treatment modality for expanded cohort. The pooled estimate represents the overall mean difference (post-treatment minus baseline), combining surgical and radiotherapeutic cohorts. Blue squares represent the point estimate for each study in the forest plot, with their size proportional to the study’s weight (i.e., its contribution to the summary estimate). Black squares represent the overall summary estimate. MD denotes the mean difference, while CL L. and CL U. denote the lower and upper limits of the confidence interval, respectively, and horizontal lines indicate 95% confidence intervals [20,21,23,24].

None of the included longitudinal studies contained an untreated or otherwise concurrent comparator group. The quantity estimated below is therefore the within-cohort change in DLQI score observed between baseline and follow-up, which cannot be attributed to treatment alone, since regression to the mean, resolution of diagnostic uncertainty and the natural course of the disease may all contribute. In the primary BCC meta-analysis, the overall mean reduction in DLQI was 2.38 points (95% CI: 0.56–4.19; *p* = 0.0101). In the expanded meta-analysis, which additionally included SCC and NMSC studies, the pooled mean reduction in DLQI was 1.90 points (95% CI: 0.84–2.96). The analyses were based on aggregated summary statistics reported in the original publications. When repeated using the actual follow-up sample sizes where these were reported, the pooled estimates were essentially unchanged (BCC-only 2.35 points, 95% CI: 0.59–4.11; expanded 1.87 points, 95% CI: 0.85–2.89, at r = 0.50), indicating that the use of baseline sample sizes did not appreciably affect the results. Heterogeneity in the pre–post analyses was likewise high (I^2^ = 93.7% for the BCC-only and 90.3% for the expanded analysis at r = 0.50), so these pooled values also summarize a dispersed rather than a homogeneous body of evidence (Figure 6 and Figure 7).

Among the individual studies, the greatest improvement in DLQI was observed in the study by Blackford et al., with a mean reduction of 4.00 points (95% CI: 2.95–5.05), whereas the smallest improvement was reported by John S. Rhee et al., with a mean reduction of 0.70 points (95% CI: 0.20–1.20) [21,24].

### 3.5. Subgroup Analysis

**Figure 8:**
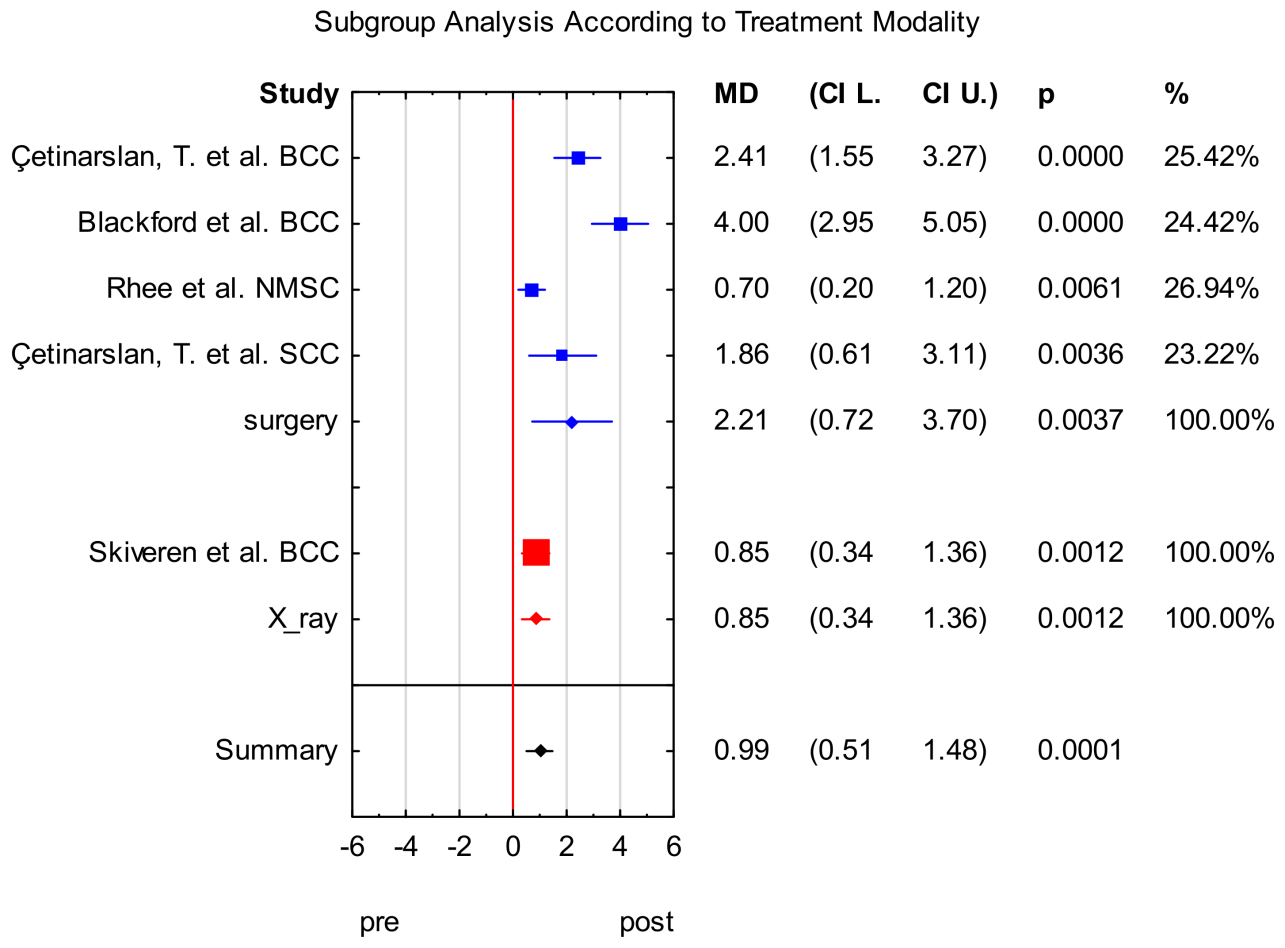
Forest plot of subgroup-stratified pre–post treatment changes in DLQI scores according to treatment modality (surgery vs. radiotherapy) using a mixed-effects meta-analytic model. The pooled estimate represents the subgroup-adjusted mean difference accounting for between-modality heterogeneity. Blue squares represent studies involving patients after surgery, while red squares represent studies involving patients after radiotherapy. The size of each square is proportional to the study’s weight (i.e., its contribution to the summary estimate). Black squares represent the overall summary estimate. MD denotes the mean difference, while CL L. and CL U. denote the lower and upper limits of the confidence interval, respectively. *p* denotes the probability value, and horizontal lines indicate 95% confidence intervals [20,21,23,24].

Subgroup analysis according to treatment modality demonstrated a pooled DLQI improvement of 2.21 points (95% CI: 0.73–3.70; *p* = 0.0037) following surgical treatment. In contrast, radiotherapy (X-ray treatment) was associated with a smaller but statistically significant improvement in DLQI scores, with a pooled mean difference of 0.85 points (95% CI: 0.34–1.36; *p* = 0.0012). As only one radiotherapy dataset was available and treatment groups were not directly comparable, this subgroup finding should be interpreted as descriptive rather than as the evidence of the superiority of one treatment modality over another. The two modality subgroups also differ in ways unrelated to the intervention itself: the radiotherapy estimate derives from a single Danish cohort of 25 patients treated with superficial X-ray therapy and assessed at three months, whereas the surgical estimate pools cohorts from Turkey and the United Kingdom with different tumor sizes, anatomical distributions and assessment intervals. Radiotherapy is moreover typically selected for patients who are older, frailer or have tumors in anatomically difficult sites, so the subgroups are not exchangeable and the difference between them may reflect case mix and timing rather than any property of the treatment. In addition, both subgroup estimates lie below the accepted minimal clinically important difference for the DLQI, so neither modality produced an average change that would be expected to be perceptible to the individual patient. Overall, these findings indicate that treatment of NMSC was followed by a measurable but small change in patient-reported quality of life, and the apparent difference between modalities should be regarded as descriptive only (Figure 8).

### 3.6. Certainty of the Evidence

The GRADE assessment is presented in Table 2. The certainty of the evidence was rated as very low for all four outcomes. Starting from low certainty because all included studies were observational, the evidence was downgraded further for risk of bias, as the studies were susceptible to selection bias and confounding, follow-up was incomplete in two studies, and none adequately adjusted for important prognostic factors such as tumor size, anatomical site, reconstruction method, or recurrent disease. The certainty was also downgraded for very serious inconsistency, because statistical heterogeneity was substantial (I^2^ approximately 90–97%), indicating considerable variability in effect estimates across studies. Imprecision was judged to be serious owing to the small number of included studies, relatively small sample sizes, and correspondingly wide confidence intervals. The expanded analyses were downgraded additionally for indirectness, because they combined BCC, SCC, and mixed NMSC populations that differ in prognosis, treatment, and cosmetic burden and therefore do not directly address a single, well-defined clinical population. Publication bias could not be formally assessed because fewer than ten studies contributed to each outcome; therefore, small-study effects cannot be excluded. Overall, the evidence should be regarded as being of very low certainty, and the findings should be interpreted as hypothesis-generating rather than confirmatory.

## 4. Discussion

The assessment of quality of life (QoL) has become increasingly important in patients treated for skin cancers because therapeutic outcomes extend beyond oncologic control and include functional, esthetic, and psychosocial consequences. Due to the anatomical and social importance of the face and neck region, treatment-related morbidity may substantially affect the patients’ daily functioning, self-image, and interpersonal relationships. Disfigurement, visible scarring, or functional impairment may contribute to emotional distress and negatively influence social, marital, and even professional interactions. Therefore, instruments evaluating the dermatology-related quality of life, such as the Dermatology Life Quality Index, are valuable tools for understanding the broader impact of treatment on the patients’ well-being.

The present meta-analysis showed that patients with non-melanoma skin cancer experienced measurable impairment in dermatology-specific quality of life before treatment, although the overall burden was relatively mild. The similar pooled estimates obtained in the primary analysis, restricted to studies including patients with basal cell carcinoma (BCC) (DLQI 3.76), and in the expanded analysis, which incorporated studies involving broader non-melanoma skin cancer populations (DLQI 4.07), indicate that broadening the population beyond BCC did not materially shift the pooled baseline value. This concordance should not, however, be described as robustness: with I^2^ exceeding 95% in both analyses, the two pooled means are summaries of highly dispersed evidence rather than estimates of a common underlying value, and their similarity reflects the fact that the same few studies dominate both analyses. Because the expanded analyses combine pure BCC, pure SCC, mixed BCC/SCC and broader NMSC cohorts—conditions that differ in prognosis, treatment strategy, anatomical distribution, cosmetic impact and psychological burden—they are explicitly exploratory, and no estimate derived from them can be taken as representative of BCC alone. Importantly, the expanded analyses including SCC and broader NMSC populations should not be interpreted as estimates of quality-of-life impairment in BCC. These analyses were conducted solely to explore whether the limited evidence base available for BCC could be contextualized within the broader group of keratinocyte cancers. However, differences in tumor biology, prognosis, treatment pathways, anatomical distribution, and cosmetic consequences substantially limit direct comparability between these populations.

Given the substantial between-study heterogeneity, we additionally performed subgroup analyses according to tumor type to explore potential sources of variability. These analyses suggested differences in baseline quality of life across diagnostic groups, with the greatest impairment observed among patients with squamous cell carcinoma (SCC), followed by those with BCC and mixed non-melanoma skin cancer populations (DLQI 6.22, 3.76 and 2.69, respectively). This pattern is clinically plausible, as SCC generally exhibits more aggressive clinical behavior, has a greater potential for local tissue invasion, and may require more extensive treatment than BCC. Nevertheless, these findings should be interpreted cautiously because the subgroup analyses were exploratory and based on a limited number of studies.

Importantly, the observed baseline impairment appeared to improve following treatment. The meta-analysis demonstrated a significant improvement in dermatology-specific quality of life after treatment, with consistent findings in both the primary analysis restricted to BCC and the expanded analysis including SCC and broader NMSC populations. Although the magnitude of improvement was somewhat lower in the expanded analysis, the direction of the treatment effect remained unchanged, suggesting that treatment-related improvement in QoL was consistent across different patient populations. Sensitivity analyses showed that the estimates were insensitive to the assumed pre–post correlation and to the choice of baseline versus follow-up sample size. This insensitivity concerns the analytical assumptions only; it does not imply agreement between studies, which remained poor, and the confidence intervals and *p*-values reported for these analyses remain conditional on an assumed rather than an observed correlation.

Substantial between-study heterogeneity persisted in the pre–post analyses, and subgroup analysis by treatment modality suggested a numerically larger change after surgery than after radiotherapy. This comparison cannot support any inference about the relative merits of the two modalities: the radiotherapy estimate derives from a single cohort of 25 patients, and radiotherapy is preferentially offered to older or frailer patients and to anatomically difficult tumors, so the subgroups differ systematically in case mix, tumor characteristics and assessment interval. The apparent difference may equally reflect the immediate removal of the visible lesion and prompt reassurance of clearance after surgery, as against the longer treatment course and delayed cosmetic result of radiotherapy. It is therefore reported as a possible contributor to heterogeneity and as a descriptive observation only.

Adequately powered comparative studies are needed to establish whether treatment modality genuinely influences quality-of-life outcomes.

The clinical relevance of the observed change must be interpreted with care. A reduction of about four points has generally been regarded as the smallest change in DLQI that patients are likely to notice, although this threshold was established mainly in chronic inflammatory skin disease and has not been validated specifically for basal cell carcinoma or other keratinocyte cancers. The pooled changes observed here 1.90 points in the expanded and 2.38 points in the BCC-only analysis lie below that threshold, and this is arguably the central finding of the present work. Statistical significance and clinical importance are distinct: the *p*-values indicate only that the average change is unlikely to be exactly zero, whereas the minimal clinically important difference indicates how large a change must be before a patient perceives it. Every subgroup estimate in this review, including the surgical subgroup, also fell below the threshold. Accordingly, although the observed changes were statistically significant, they did not exceed the accepted threshold for a clinically meaningful improvement in DLQI and therefore cannot be interpreted as demonstrating a perceptible improvement in patient-reported quality of life on average. This does not mean that treatment lacks value: the low baseline scores leave little room for measurable improvement, and a generic instrument may be insensitive to the appearance-, scar- and recurrence-related concerns that matter most to these patients. It does mean that the DLQI, used alone, does not demonstrate a clinically important benefit, and that disease-specific instruments are required to establish whether one exists.

The relatively low baseline DLQI scores were also not unexpected. Basal cell carcinoma usually grows slowly, causes few physical symptoms, and has an excellent prognosis. As a result, many patients report only a limited impact on their quality of life before treatment, leaving less room for measurable improvement after therapy.

Baseline DLQI scores were also lower than those typically reported in melanoma, psoriasis, or atopic dermatitis. This is consistent with the different clinical course of these diseases. Melanoma is often associated with greater anxiety because of the risk of progression and recurrence, whereas psoriasis and atopic dermatitis are chronic conditions that can affect daily life for many years. In contrast, the burden of basal cell carcinoma is more often related to the visibility of the lesion, its location, and the cosmetic consequences of treatment than to persistent physical symptoms.

The observed heterogeneity among studies may be related to differences in tumor location, treatment modality, patient age, and baseline psychosocial characteristics. Future studies should incorporate standardized reporting of clinical characteristics and longer follow-up to better identify factors influencing functional and psychosocial outcomes. In addition, part of the quality-of-life burden in these patients may arise from treatment-related factors, including the extent of surgical defects and reconstructive requirements. Improved preoperative assessment of tumor characteristics may support treatment planning and help minimize unnecessary tissue removal, particularly in cosmetically sensitive areas. Similarly, OCT-based preoperative margin mapping aims to reduce surgical stages, limit the extent of excision defects, and improve procedural efficiency [26]. A recent prospective study suggested that preoperative ultrasound assessment may improve estimation of BCC depth and support surgical planning [27]. Although these approaches have not yet been directly linked to patient-reported outcomes, better preoperative tumor characterization may contribute to reducing treatment-related burden.

Overall, the findings of this meta-analysis emphasize that the quality of life should be considered an integral component of treatment evaluation in patients with cervicofacial NMSCs. In addition to achieving oncological cure, clinicians should aim to minimize functional impairment and optimize cosmetic outcomes to improve the patients’ overall well-being and social functioning.

Although baseline DLQI scores suggested only mild impairment, visible lesions and treatment-related consequences may still affect patients’ daily lives and psychosocial well-being.

The improvement in the post-treatment QoL further supports the evolving perspective that therapeutic success in NMSC should not be assessed solely based on local tumor control or recurrence rates. Oncologic practice increasingly emphasizes patient-centered outcomes, particularly in older populations, where maintaining independence, preserving social functioning, and minimizing treatment-related disruption may be equally important as oncologic efficacy itself. In this setting, visible lesions affecting the head and neck region are widely recognized as factors capable of negatively influencing self-esteem, body image, interpersonal relationships, and emotional well-being, even in tumors characterized by relatively indolent biological behavior [28,29].

Rapid removal of visible lesions and shorter recovery may reduce treatment burden and improve patient satisfaction, particularly in older adults and in patients with tumors located in cosmetically sensitive areas [30]. However, comparable oncologic outcomes do not necessarily translate into the same patient experience. In facial non-melanoma skin cancers, quality of life is also influenced by cosmetic appearance, scar perception, recovery, fear of recurrence, tumor location, defect size, and patient characteristics, all of which contribute to postoperative satisfaction beyond procedural success alone [31]. These observations emphasize the importance of incorporating patient-reported outcomes alongside traditional oncologic endpoints when selecting treatment for NMSC.

These findings underline the importance of including patient-reported outcome measures when evaluating treatment for non-melanoma skin cancer. Disease-specific PROMs may provide a better understanding of treatment burden, cosmetic outcomes, and patient satisfaction and should be considered in future prospective studies [28,31].

The perception of disease burden also appears to vary considerably across age groups Previous studies suggest that younger patients may experience greater distress related to cosmetic outcomes and appearance-related concerns, whereas older adults may report a comparatively lower psychosocial impact from BCC despite similar disease characteristics [28,30]. This observation emphasizes that individualized treatment planning should consider not only tumor biology and procedural feasibility but also patient expectations, lifestyle, and psychosocial priorities.

Several limitations of the present meta-analysis should also be acknowledged. First, only one included study evaluated quality-of-life outcomes following radiotherapy. Consequently, the subgroup analysis concerning radiotherapeutic treatment should be interpreted with caution, as the available evidence remains limited. Larger cohorts of patients treated with radiotherapy are needed before any conclusion can be drawn about the comparative impact of different treatment modalities on DLQI outcomes.

Another important limitation was the inability to perform subgroup analyses according to clinically relevant tumor characteristics, including anatomical location, tumor size, lesion duration, and primary versus recurrent disease. Although the total number of participants across included studies was not negligible, the limited number of independent studies remained a major constraint, reducing the precision of pooled estimates and precluding reliable exploration of potential moderators of between-study heterogeneity. These factors are likely to influence patients’ quality of life and may represent important sources of variability between studies. However, the included studies reported these variables inconsistently and used different classification systems. For example, the study by John S. Rhee et al. categorized lesions according to detailed anatomical regions, including the nose, lips, eyelids, ears, forehead, temple, neck, scalp, and other locations, while also reporting lesion size and previous treatment status. In contrast, the study by Robabeh Abedini et al. used broader anatomical categories, such as the face, head and neck, trunk and extremities, or combined anatomical regions, and did not report longitudinal quality-of-life outcomes. Similarly, Çetinarslan et al. [20] reported lesion duration and anatomical location but used different anatomical classifications and did not provide lesion size data. Because of these differences in study design, reporting methods, and variable definitions, pooled subgroup analyses or meta-regression according to these clinically relevant characteristics were not feasible. These inconsistencies likely contributed to the substantial between-study heterogeneity observed in the present meta-analysis and should be considered when interpreting the pooled estimates. Future studies should adopt standardized reporting of tumor characteristics and patient-reported outcomes to facilitate more robust and clinically informative evidence synthesis. Moreover, formal assessment of publication bias was not possible. With fewer than ten included studies, funnel plots and associated statistical tests have insufficient power and were therefore not performed; consequently, small-study effects and publication bias cannot be excluded.

Although the DLQI remains one of the most widely used dermatologic quality-of-life instruments, it may not fully capture psychosocial dimensions specific to keratinocyte cancers, particularly when lesions occur in cosmetically sensitive facial regions. This limitation may be further compounded by the lack of sex-specific analyses, as most included studies did not report DLQI outcomes stratified by sex. Given that psychosocial perceptions, cosmetic concerns, and social functioning may differ between male and female patients, future studies should provide more detailed demographic and subgroup data and consider the use of disease-specific quality-of-life instruments. Such approaches would enable a more comprehensive assessment of the factors influencing the quality of life in patients with non-melanoma skin cancer.

## 5. Conclusions

The findings indicate that non-melanoma skin cancers, including basal cell carcinoma and squamous cell carcinoma, may negatively influence dermatology-related quality of life, as captured by the DLQI, prior to treatment, despite their relatively favorable oncologic prognosis. All conclusions below refer specifically to the DLQI, because the quantitative synthesis was confined to this generic instrument and because the analyses pooling BCC with SCC and broader NMSC cohorts are exploratory, they should not be extrapolated to quality of life as assessed by disease-specific measures, nor to BCC considered in isolation.

A statistically significant reduction in DLQI scores was observed between baseline and follow-up. Because none of the included studies had a comparator group, this represents a change following treatment rather than an established treatment effect. The reduction was modest and did not reach the published threshold for a minimal clinically important difference, so on average, it would not be expected to be perceptible to the individual patient. The magnitude of improvement appeared numerically greater after surgical treatment; however, the available evidence is insufficient to determine whether treatment modality independently influences QoL outcomes. These changes in the quality of life should be considered an important complementary measure of treatment success alongside recurrence rates, margin status, and procedural safety.

The burden associated with NMSC extends beyond physical manifestations alone and may include emotional distress, appearance-related concerns, and impaired social functioning, particularly in patients with lesions located in visible anatomical areas. Treatment planning should increasingly incorporate patient expectations, cosmetic outcomes, treatment-related burden, and long-term satisfaction in addition to conventional oncologic considerations.

Integrating disease-specific quality-of-life instruments into future prospective studies and routine clinical practice may support more individualized therapeutic strategies and enable a broader assessment of treatment effectiveness in patients with keratinocyte cancers.

## Figures and Tables

**Table 1:** Sensitivity analysis of the standardized mean change (Hedges’ g, corrected for small-sample bias) according to the assumed pre–post correlation coefficient r. Positive values denote improvement (reduction) in DLQI score. Hedges’ g is reported here to place cohorts of differing size on a common scale; all pooled treatment estimates in the text are expressed as raw mean differences in DLQI points.

Study (Cohort)	Hedges’ g (r = 0.25)	Hedges’ g (r = 0.50)	Hedges’ g (r = 0.75)
Çetinarslan T et al. BCC [20]	0.34	0.41	0.57
Skiveren J. et al. [23]	0.53	0.62	0.80
Blackford S. et al. [24]	0.96	1.11	1.36
Çetinarslan T et al. [20] SCC	0.26	0.32	0.45
Rhee J. et al. [21]	0.20	0.25	0.35

**Table 2:** GRADE assessment of the certainty of the evidence.

Outcome	Studies (Cohorts); Patients	Study Design	Risk of Bias	Inconsistency	Indirectness	Imprecision	Publication Bias	Certainty
Baseline DLQI—BCC (primary analysis)	5 (5); 326	Observational	Serious a	Very serious b	Not serious	Serious d	Not assessable e	⊕○○○ Very low
Baseline DLQI—expanded (BCC, SCC and mixed NMSC)	6 (9); 548	Observational	Serious a	Very serious b	Serious c	Serious d	Not assessable e	⊕○○○ Very low
Change in DLQI after treatment—BCC	3 (3); 243	Uncontrolled before–after	Serious a	Very serious b	Not serious	Serious d	Not assessable e	⊕○○○ Very low
Change in DLQI after treatment—expanded	4 (5); 445	Uncontrolled before–after	Serious a	Very serious b	Serious c	Serious d	Not assessable e	⊕○○○ Very low

a Downgraded one level: observational studies were susceptible to selection bias and confounding; follow-up was incomplete in two studies, and none adequately adjusted for important prognostic factors such as tumor size, anatomical site, reconstruction method, or recurrent disease. b Downgraded two levels: substantial statistical heterogeneity (I^2^ approximately 90–97%), indicating considerable between-study variability. c Downgraded one level: pooled population combines BCC, SCC, and mixed NMSC cohorts with differing clinical characteristics, treatments, and prognoses. d Downgraded one level: few studies with relatively small sample sizes and correspondingly wide confidence intervals. e Publication bias could not be formally assessed because fewer than ten studies contributed to each outcome; therefore, small-study effects cannot be excluded. ⊕○○○, very low certainty.

**Table 3:** Risk-of-bias assessment of the included studies.

Study	Design	Risk-of-Bias Tool	Overall Risk of Bias
Rhee et al. [21]	Prospective cohort	JBI Cohort Checklist	Low
Abedini et al. [22]	Cross-sectional	JBI Analytical Cross-sectional Checklist	Moderate
Skiveren et al. [23]	Prospective cohort	JBI Cohort Checklist	Low–moderate
Çetinarslan et al., 2020 [20]	Prospective cohort	JBI Cohort Checklist	Moderate
Finlay et al. [19]	DLQI validation study	COSMIN Risk of Bias Checklist	Low–moderate
Blackford et al. [24]	Prospective cohort	JBI Cohort Checklist	moderate

**Table 4:** Characteristics and quality-of-life outcomes of the included studies. Patient numbers are presented for each study and tumor category; the final row summarizes the overall number of participants across the six included studies. Tumor categories are not mutually exclusive because some patients had both BCC and SCC. BCC, basal cell carcinoma; SCC, squamous cell carcinoma; NMSC, non-melanoma skin cancer; DLQI, Dermatology Life Quality Index.

Study 1994	Country	Patients, *n*	Tumor Type (*n*)	Age, Years	Sex (F/M)	Baseline DLQI, Mean ± SD	Post-Treatment DLQI, Mean ± SD (Timepoint; *n*)
Finlay et al., 1994 [19]	UK	8	BCC (8)	60.6	5/3	2.0 ± 2.2	Not reported
Blackford et al., 1996 [24]	UK	44	BCC (44)	65 (35–81)	Not reported	5.3 ± 4.1	8.7 ± 9.6 (1–2 weeks; *n* = 44); 1.3 ± 2.1 (3 months; *n* = 34)
Rhee et al., 2004 [21]	USA	121	BCC (103), SCC (16), other (2)	62.17 ± 15.4	65/56	2.40 ± 2.7	1.7 ± 2.9 (4 months; *n* = 101)
Skiveren et al., 2012 [23]	Denmark	25	BCC (25); X-ray therapy	69.5 ± 11.5	9/16	1.2 ± 1.5	0.36 ± 0.9 (3 months; *n* = 25)
Abedini et al., 2019 [22]	Iran	95	BCC (75), SCC (15), Both BCC and SCC (5)	64.6 ± 2.5	21/74	BCC 3.9 ± 0.4; SCC 5.7 ± 5.2; BCC + SCC 4.8 ± 4.7; all NMSC 4.1 ± 4.25	Not reported
Çetinarslan et al., 2020 [20]	Turkey	255	BCC (174), SCC (81)	BCC 63.07 ± 15.62 (32–90); SCC 64.95 ± 13.37 (42–92)	113/142	BCC 6.37 ± 6.28; SCC 6.35 ± 6.16	BCC 3.96 ± 5.14; SCC 4.49 ± 5.24 (3 months; *n* = 255)
Total	–	548	BCC 429, SCC112, Both BCC and SCC (5) other (2)	–	–	–	–

## Data Availability

All data supporting the findings of this study are derived from publicly available published literature and are included within the article and its Supplementary Materials.

## Supplementary Materials

The following supporting information can be downloaded at: https://www.mdpi.com/article/10.3390/jcm15166351/s1, Table S1. Complete search strategies for all databases, Table S2. Detailed risk-of-bias assessment of included studies, Table S3. PRISMA 2020 Checklist.
