# Supplementary material for: Dermatology-Related Quality of Life Measured with the Dermatology Life Quality Index in Basal Cell Carcinoma and Other Non-Melanoma Skin Cancers: A Systematic Review and Exploratory Meta-Analysis
Source: J Clin Med. 2026 Aug 17;15(16):6351. doi: 10.3390/jcm15166351 (PMC13513170; doi:10.3390/jcm15166351)
Supplement: Supplementary file 1 [file jcm-15-06351-s001.zip › jcm-4440515-supplementary.pdf]

The keywords used for the search were the terms *"basal cell carcinoma", "BCC", "non-melanoma skin cancer", "NMSC"*, combined with "quality of life", "QoL", "Dermatology Life Quality Index", and "DLQI".

Database	Complete search strategy	Search date
PubMed	("Basal Cell Carcinoma"[Mesh Terms] OR "basal cell carcinoma"[tiab] OR BCC[tiab] OR "non-melanoma skin cancer"[tiab] OR NMSC[tiab] OR "Carcinoma, Squamous Cell"[Mesh Terms] OR "squamous cell carcinoma"[tiab] OR SCC[tiab]) AND ("Quality of Life"[Mesh Terms] OR "quality of life"[tiab] OR QoL[tiab] OR "Dermatology Life Quality Index"[tiab] OR DLQI[tiab])	5 April 2026
Scopus	TITLE-ABS-KEY("basal cell carcinoma" OR BCC OR "non-melanoma skin cancer" OR NMSC OR "squamous cell carcinoma" OR SCC) AND TITLE-ABS-KEY("quality of life" OR QoL OR "Dermatology Life Quality Index" OR DLQI)	5 April 2026
Web of Science	TS=("basal cell carcinoma" OR BCC OR "non-melanoma skin cancer" OR NMSC OR "squamous cell carcinoma" OR SCC) AND TS=("quality of life" OR QoL OR "Dermatology Life Quality Index" OR DLQI)	5 April 2026

Table S1. Complete search strategies for all databases

Manual search: The reference lists of all included studies and relevant review articles were screened manually to identify additional eligible studies.

Study	Design	Risk-of-bias tool	Q1	Q2	Q3	Q4	Q5	Q6	Q7	Q8	Q9	Q10	Overall risk of bias
Rhee et al. [21]	Prospective cohort	JBI Cohort Checklist	Yes	Yes	Yes	Yes	Yes	Unclear	Yes	Yes	Yes	Yes	Low
Abedini et al. [22]	Cross-sectional	JBI Analytical Cross-sectional Checklist	Yes	Yes	Yes	Yes	Yes	not applicable	Yes	Yes	Yes	not applicable	Moderate
Skiveren et al. [23]	Prospective cohort	JBI Cohort Checklist	Yes	Yes	Yes	Yes	Yes	Unclear	Yes	Yes	Yes	Yes	Low–moderate
Çetinarslan et al. (2020) [20]	Prospective cohort	JBI Cohort Checklist	Yes	Yes	Yes	Yes	Yes	Yes	Yes	Yes	Unclear	Yes	Moderate
Finlay et al. [19]	DLQI validation study	COSMIN Risk of Bias Checklist	Yes	Yes	Yes	Yes	Yes	not applicable	not applicable	not applicable	not applicable	not applicable	Low–moderate
Blackford et al. [24]	Prospective cohort	JBI Cohort Checklist	Yes	Yes	Yes	Unclear	Unclear	Yes	Yes	Yes	No	Yes	Moderate

Table S2. Detailed risk-of-bias assessment of included studies

JBI Cohort Checklist domains:

Q1. Were the two groups similar and recruited from the same population?
Q2. Were the exposures measured similarly to assign people to both exposed and unexposed groups?
Q3. Was the exposure measured in a valid and reliable way?
Q4. Were confounding factors identified?
Q5. Were strategies to deal with confounding factors stated?
Q6. Were the participants free of the outcome at the start of the study?
Q7. Were the outcomes measured in a valid and reliable way?
Q8. Was the follow-up time reported and sufficient to be long enough for outcomes to occur?
Q9. Was follow-up complete, and if not, were the reasons for loss to follow-up described and explored?
Q10. Were strategies to address incomplete follow-up utilized?

COSMIN domains:

Q1. Was the study population adequately described and representative of the target population?
Q2. Was the measurement instrument adequately described?
Q3. Were appropriate methods used to evaluate reliability?

Q4. Were appropriate methods used to evaluate validity?
Q5. Were statistical analyses appropriate for assessing measurement properties

Table S3. PRISMA 2020 Checklist

Section and Topic	Item #	Checklist item	Location where item is reported
**TITLE**			
Title	1	Identify the report as a systematic review.	page 1
**ABSTRACT**			
Abstract	2	See the PRISMA 2020 for Abstracts checklist.	page 1
**INTRODUCTION**			
Rationale	3	Describe the rationale for the review in the context of existing knowledge.	pages 2
Objectives	4	Provide an explicit statement of the objective(s) or question(s) the review addresses.	page 3
**METHODS**			
Eligibility criteria	5	Specify the inclusion and exclusion criteria for the review and how studies were grouped for the syntheses.	pages 3-4
Information sources	6	Specify all databases, registers, websites, organisations, reference lists and other sources searched or consulted to identify studies. Specify the date when each source was last searched or consulted.	page 3
Search strategy	7	Present the full search strategies for all databases, registers and websites, including any filters and limits used.	page 3
Selection process	8	Specify the methods used to decide whether a study met the inclusion criteria of the review, including how many reviewers screened each record and each report retrieved, whether they worked independently, and if applicable, details of automation tools used in the process.	page 3
Data collection process	9	Specify the methods used to collect data from reports, including how many reviewers collected data from each report, whether they worked independently, any processes for obtaining or confirming data from study investigators, and if applicable, details of automation tools used in the process.	page 3
Data items	10a	List and define all outcomes for which data were sought. Specify whether all results that were compatible with each outcome domain in each study were sought (e.g. for all measures, time points, analyses), and if not, the methods used to decide which results to collect.	pages 3-4
	10b	List and define all other variables for which data were sought (e.g. participant and intervention characteristics, funding sources). Describe any assumptions made about any missing or unclear information.	pages 3-4
Study risk of bias assessment	11	Specify the methods used to assess risk of bias in the included studies, including details of the tool(s) used, how many reviewers assessed each study and whether they worked independently, and if applicable, details of automation tools used in the process.	page 5
Effect measures	12	Specify for each outcome the effect measure(s) (e.g. risk ratio, mean difference) used in the synthesis or presentation of results.	pages 5

Section and Topic	Item #	Checklist item	Location where item is reported
Synthesis methods	13a	Describe the processes used to decide which studies were eligible for each synthesis (e.g. tabulating the study intervention characteristics and comparing against the planned groups for each synthesis (item #5)).	pages 5-6
	13b	Describe any methods required to prepare the data for presentation or synthesis, such as handling of missing summary statistics, or data conversions.	
	13c	Describe any methods used to tabulate or visually display results of individual studies and syntheses.	page 5
	13d	Describe any methods used to synthesize results and provide a rationale for the choice(s). If meta-analysis was performed, describe the model(s), method(s) to identify the presence and extent of statistical heterogeneity, and software package(s) used.	pages 6-8,
	13e	Describe any methods used to explore possible causes of heterogeneity among study results (e.g. subgroup analysis, meta-regression).	Page 9
	13f	Describe any sensitivity analyses conducted to assess robustness of the synthesized results.	pages 9
Reporting bias assessment	14	Describe any methods used to assess risk of bias due to missing results in a synthesis (arising from reporting biases).	pages 11–12
Certainty assessment	15	Describe any methods used to assess certainty (or confidence) in the body of evidence for an outcome.	
**RESULTS**			
Study selection	16a	Describe the results of the search and selection process, from the number of records identified in the search to the number of studies included in the review, ideally using a flow diagram.	pages 5-8
	16b	Cite studies that might appear to meet the inclusion criteria, but which were excluded, and explain why they were excluded.	page 4
Study characteristics	17	Cite each included study and present its characteristics.	Pages 4–7
Risk of bias in studies	18	Present assessments of risk of bias for each included study.	pages 9-11
Results of individual studies	19	For all outcomes, present, for each study: (a) summary statistics for each group (where appropriate) and (b) an effect estimates and its precision (e.g. confidence/credible interval), ideally using structured tables or plots.	pages 11-13
Results of syntheses	20a	For each synthesis, briefly summarise the characteristics and risk of bias among contributing studies.	pages 8-9
	20b	Present results of all statistical syntheses conducted. If meta-analysis was done, present for each the summary estimate and its precision (e.g. confidence/credible interval) and measures of statistical heterogeneity. If comparing groups, describe the direction of the effect.	Pages 6-11
	20c	Present results of all investigations of possible causes of heterogeneity among study results.	pages 9-10
	20d	Present results of all sensitivity analyses conducted to assess the robustness of the synthesized results.	pages 8-11
Reporting biases	21	Present assessments of risk of bias due to missing results (arising from reporting biases) for each synthesis assessed.	pages 9–11
Certainty of evidence	22	Present assessments of certainty (or confidence) in the body of evidence for each outcome assessed.	–

Section and Topic	Item #	Checklist item	Location where item is reported
**DISCUSSION**			
Discussion	23a	Provide a general interpretation of the results in the context of other evidence.	pages 13–14
	23b	Discuss any limitations of the evidence included in the review.	pages 15–16
	23c	Discuss any limitations of the review processes used.	pages 15–16
	23d	Discuss implications of the results for practice, policy, and future research.	pages 15–17
**OTHER INFORMATION**			
Registration and protocol	24a	Provide registration information for the review, including register name and registration number, or state that the review was not registered.	the review was not registered
	24b	Indicate where the review protocol can be accessed, or state that a protocol was not prepared.	protocol was not prepared
	24c	Describe and explain any amendments to information provided at registration or in the protocol.	-
Support	25	Describe sources of financial or non-financial support for the review, and the role of the funders or sponsors in the review.	not applicable
Competing interests	26	Declare any competing interests of review authors.	no interests to declare
Availability of data, code and other materials	27	Report which of the following are publicly available and where they can be found: template data collection forms; data extracted from included studies; data used for all analyses; analytic code; any other materials used in the review.	all articles which were used for the meta-analysis are available on databases.
